# Advances in Wine Physicochemical Properties, Sensory Attributes, and Health Benefits

**DOI:** 10.3390/foods14223889

**Published:** 2025-11-14

**Authors:** Irena Budić-Leto, Jasenka Gajdoš Kljusurić

**Affiliations:** 1 Institute for Adriatic Crops and Karst Reclamation, 21000 Split, Croatia; 2 Faculty of Food Technology and Biotechnology, University of Zagreb, Pierottijeva 6, 10000 Zagreb, Croatia; jasenka.gajdos@pbf.unizg.hr

## 1. Introduction

Wine is one of the most studied fermented beverages in the world, representing a unique intersection of tradition, culture, and modern science [1,2]. Its complexity arises from the interaction of grape composition, *terroir*, microorganisms, and winemaking techniques [3,4], while its potential health effects continue to attract the interest of researchers and consumers alike [5]. Over the past decades, scientific advances have transformed our understanding of wine, moving beyond simple chemical characterisation toward a multi-dimensional approach that integrates viticulture, oenology, food technology, health sciences, and consumer behaviour [6,7].

This book edition brings together the articles published in the *Foods* Special Issue “Advances in Wine Physicochemical Properties, Sensory Attributes, and Health Benefits,” conceived as a continuation of the successful first edition, “Grape Wine: Physicochemical Properties, Sensory Attributes, and Health Benefits.” Its purpose is to provide readers with an integrated perspective on the latest research into wine composition, sensory quality, and potential health-promoting properties.

The collected contributions reflect the diversity and innovation of contemporary wine science. They explore a wide spectrum of topics—from the influence of vineyard environment and winemaking techniques on the aromatic and phenolic profile of wines, to the application of advanced analytical techniques such as non-destructive spectroscopy and chemometric modelling. Special attention is given to understanding the compounds responsible for physicochemical characteristics, sensory perception, and antioxidant activity, thereby supporting the view of wine as a potential functional food [8]. Additionally, these studies examine the role of microorganisms, the impact of technological innovations, and consumer perception, thereby opening up new opportunities for the development of wines tailored to evolving market demands and health-conscious lifestyles.

## 2. Overview of the Contributions

The opening chapter examines how different alcoholic bases affect the flavour and sensory balance of soaked greengage wine, combining chromatographic analyses with trained sensory evaluation to optimize fruit wine production (contribution 1). The study concludes that the choice of base liquor significantly influences the flavour profile of soaked greengage wine, with the 50% edible alcohol treatment performing best overall, providing practical guidance for fruit wine production and optimisation.

Furthermore, many factors influence grape and wine quality. The second study provides a detailed *terroir* and vintage analysis of Cabernet Sauvignon, linking soil composition, climatic variation, and vineyard location with key aroma compounds and wine style (contribution 2). The findings indicate that within a single slope, *terroir* (soil and plot) plus vintage interact to produce noticeable differences in aroma profile and wine style, and that understanding these relationships can help refine vineyard management to improve wine aroma quality.

Natural additives are increasingly used in winemaking, with many commercial products claiming diverse technological and sensory benefits. Another contribution investigates the influence of oenological additives and individual taster phenotypes on flavour persistence, demonstrating how compounds such as tannins and mannoproteins can modulate astringency and retronasal aroma (contribution 3). The results of this research highlight that both individual taste phenotype (PROP taster vs. non-taster) and choice of oenological additive have measurable impacts on how long flavour (especially astringency and certain aroma attributes) lingers in wine, suggesting these tools can be used strategically to modulate wine sensory profile.

Wine contains a wide array of chemical compounds, attracting researchers eager to explore and understand its intricate composition. Subsequent research has highlighted innovative vinification techniques in Teran wine, demonstrating how extended maceration and thermal pre-treatments can enhance phenolic complexity and sensory quality (contribution 4). Overall, the results indicate that using non-standard vinification techniques can significantly boost the bioactive composition and improve the sensory quality of Teran wine, adding potential market value.

Several chapters focus on enhancing the bioactive and antioxidant potential of wines, including studies on melatonin addition during fermentation (contribution 5) and the selection of indigenous *Saccharomyces cerevisiae* strains to preserve polyphenols and improve functional value (contribution 6). Eremia and coworkers examined the impact of melatonin addition and different maceration techniques (punch-down and pump-over) on the polyphenolic profile and antioxidant activity of Feteasca Neagra and Cabernet Sauvignon wines. Early addition of melatonin during fermentation proved to be an effective tool for enriching wines with antioxidant compounds, though further studies are needed to confirm the stability and reproducibility of these effects during ageing. Romano and collaborators aimed to identify yeast strains that minimise the adsorption of polyphenolic compounds during fermentation, thereby preserving the antioxidant capacity of the wine. A total of 136 yeast strains were analysed through micro-scale fermentations, assessing their impact on total phenolic content and antioxidant capacity. These findings suggest that the selected indigenous yeast strains can be utilized as functional fermentation starters to produce red wines with enriched polyphenolic content, aligning with the growing consumer interest in wines with potential health benefits. This research represents a significant step towards the development of “functional” fermentation starters, offering a biotechnological approach to enhance the nutritional and sensory quality of red wines.

Sustainable approaches to winemaking are also represented through the valorization of Malbec and Torrontés pomace, revealing their rich phenolic content and potential in food, cosmetic, and pharmaceutical industries (contribution 7). Results showed that Malbec pomace had much higher total phenolics and flavonoids compared to Torrontés, and superior antioxidant, reducing, and radical-scavenging activities. The extracts showed no acute toxicity in several models, but Torrontés extract at high concentration reduced the viability of certain intestinal cell lines. Both extracts inhibited lipoxygenase, and Malbec also inhibited tyrosinase effectively.

Novel yeast strategies are presented in research on non-*Saccharomyces* species such as *Lachancea thermotolerans* and *Torulaspora delbrueckii*, showing their capacity to modulate acidity, aromatic complexity, and sensory appeal in red wines (contribution 8). Overall, *Torulaspora delbrueckii* × *S. cerevisiae* proved most promising for improving the aromatic complexity and sensory appeal of Babić wines, though further large-scale studies are needed.

In response to climate-driven challenges, Canonico and coworkers (contribution 9) explore ethanol reduction through sequential fermentation with *Starmerella bombicola*, demonstrating improved balance and mouthfeel. Compared to pure *S. cerevisiae* fermentation, the sequential approach lowered ethanol by about 0.8–1% *v*/*v* and increased glycerol by 50%, improving wine body and structure. It also enhanced lactic acid and specific aromatic notes, giving wines superior sensory characteristics.

Food safety and quality are addressed through the evaluation of grape pomace as a natural alternative for removing ochratoxin A (OTA) while preserving beneficial metabolites, compared to conventional activated carbon treatment (contribution 10). Importantly, pomace repassage effectively reduced OTA without significantly compromising wine quality. These results suggest grape pomace represents a promising natural adsorbent for OTA decontamination in winemaking.

Finally, the collection concludes with an analysis of consumer perception of moderate wine consumption, highlighting the role of health-related label information and providing insights for clearer communication and the development of functional wine products (contribution 11). Findings suggest that wine labels play a critical role in shaping consumer awareness, highlighting the need for clearer communication about moderation, as well as opportunities for developing functional wines with added health value.

## 3. Conclusions

The contributions assembled in this volume illustrate the remarkable breadth and dynamism of current wine research. Collectively, they show how advances in viticulture, oenology, and analytical science are converging to improve our understanding of wine’s chemical complexity, sensory diversity, and potential health benefits. From exploring *terroir*-driven aroma expression and innovative vinification techniques, to selecting tailored yeast strains and employing sustainable by-product valorization, the studies presented here point toward a more knowledge-based, innovative, and responsible wine industry.

Taken together, these studies highlight the potential to produce wines that are not only sensorially refined and authentic to their origin, but also technologically optimized and aligned with modern nutritional and sustainability goals. We hope that this collection will inspire further interdisciplinary collaboration and innovative research, supporting the continued evolution of wine science and contributing to a future where wine is better understood, appreciated, and responsibly enjoyed.

## Data Availability

Not applicable.

## References

[1] Ribéreau-Gayon P., Glories Y., Maujean A., Dubourdieu D. (2006). The Microbiology of Wine and Vinifications. Handbook of Enology.

[2] Jackson R.S. (2008). Wine Science: Principles and Applications.

[3] Chalvantzi I., Banilas G., Tassou C., Nisiotou A. (2021). Biogeographical Regionalization of Wine Yeast Communities in Greece and Environmental Drivers of Species Distribution at a Local Scale. Front. Microbiol..

[4] Škrab D., Sivilotti P., Comuzzo P., Voce S., Cisilino D., Carlin S., Arapitsas P., Masuero D., Vrhovsek U. (2024). Influence of Harvest Date on Multi-Targeted Metabolomic Profile and Sensory Attributes of Ribolla Gialla Base and Sparkling Wines. OENO One.

[5] Ćorković I., Pichler A., Šimunović J., Kopjar M. (2024). A Comprehensive Review on Polyphenols of White Wine: Impact on Wine Quality and Potential Health Benefits. Molecules.

[6] Boban D., Grković I., Dželalija A.M., Gujinović D., Mudnić I., Boban M. (2025). The Effects of White Wine and Ethanol Consumption on the Proliferative Phase of Repair after a Surgically Induced Myocardial Infarction in Rats. Nutrients.

[7] de Sousa Dias A.L., Guittin-Leignadier C., Roy A., Sachot S., Maçna F., Flores D., Meudec E., Boulet J.-C., Sommerer N., Roland A. (2025). Oenological Performances of New White Grape Varieties. Beverages.

[8] Giovinazzo G., Grieco F., Grumezescu A.M., Holban A.M. (2019). Tapping into Health: Wine as Functional Beverage. Alcoholic Beverages.

